# Coronary Computed Tomography Angiographic Predictors of Non-culprit Territory Unrecognized Myocardial Infarction Assessed by Cardiac Magnetic Resonance in Non-ST-elevation Acute Coronary Syndrome

**DOI:** 10.3389/fcvm.2021.825523

**Published:** 2022-01-31

**Authors:** Kazuki Matsuda, Masahiro Hoshino, Yoshihisa Kanaji, Tomoyo Sugiyama, Toru Misawa, Masahiro Hada, Tatsuhiro Nagamine, Kai Nogami, Kodai Sayama, Yun Teng, Hiroki Ueno, Taishi Yonetsu, Tetsuo Sasano, Tsunekazu Kakuta

**Affiliations:** ^1^Division of Cardiovascular Medicine, Tsuchiura Kyodo General Hospital, Ibaraki, Japan; ^2^Department of Cardiovascular Medicine, Tokyo Medical and Dental University, Tokyo, Japan

**Keywords:** acute coronary syndrome, percutaneous coronary intervention, unrecognized myocardial infarction, cardiac magnetic resonance imaging, coronary computed tomography angiography

## Abstract

**Objectives:**

This study sought to assess the predictors of coronary computed tomography angiographic findings for non-infarct-related (non-IR) territory unrecognized myocardial infarction (UMI) in patients with a first episode of non-ST-elevation acute coronary syndrome (NSTE-ACS).

**Background:**

UMI detected by cardiac magnetic resonance imaging (CMR) is associated with adverse outcomes in patients with both acute coronary syndrome and chronic coronary syndrome. However, the association between the presence of UMI and coronary computed tomography angiographic (CCTA) findings remains unknown.

**Methods:**

We investigated 158 patients with a first clinical episode of NSTE-ACS, who underwent pre-PCI 320-slice CCTA and uncomplicated urgent percutaneous coronary intervention (PCI) within 48 h of admission. In these patients, post-PCI CMR was performed within 30 days from urgent PCI and before non-IR lesion staged PCI. UMI was assessed using late gadolinium enhancement (LGE)-CMR by identifying regions of hyperenhancement with an ischemic distribution pattern in non-IR territories (non-IR UMI). CCTA analysis included qualitative and quantitative assessments of the culprit segment, Agatston score, mean peri-coronary fat attenuation index (FAI), epicardial fat volume (EFV) and epicardial fat attenuation (EFA).

**Results:**

Non-IR UMI was detected in 30 vessel territories (9.7%, 30/308 vessels) of 28 patients (17.7%, 28/158 patients). The presence of low-attenuation plaque, spotty calcification, napkin ring sign, and positive remodeling was not significantly different between vessels with and without subtended non-IR UMI. Agatston score >30.0 (OR: 8.39, 95% confidence interval (CI): 2.17 to 32.45, *p* = 0.002), mean FAI >-64.3 (OR: 3.23, 95% CI: 1.34 to 7.81, *p* = 0.009), and stenosis severity (OR: 1.04, 95% CI: 1.02 to 1.06, *p* < 0.001) were independently associated with non-IR UMI. Neither EFV (*p* = 0.340) nor EFA (*p* = 0.700) was associated with non-IR UMI.

**Conclusion:**

The prevalence of non-IR UMI was 17.7 % in patients with first NSTE-ACS presentation. Agatston score, mean FAI, and coronary stenosis severity were independent CCTA predictors of the presence of non-IR UMI. The integrated CCTA assessment may help identify the presence of non-IR UMI before urgent PCI.

## Introduction

A large proportion of acute myocardial infarction (MI) is asymptomatic or atypically presented without clinical recognition (1–3). Unrecognized myocardial infarction (UMI) has been reported to constitute up to more than 50% of all MI in the general population and in the cohort older than 60 years or in patients with chronic coronary syndrome, depending on the cardiovascular risk and modalities to detect UMI (4, 5). Recently, the presence of unrecognized myocardial scar detected by late gadolinium enhancement (LGE) in patients presenting with the first acute AMI has been reported to be associated with worse outcomes (6, 7). The prevalence of UMI in these studies were 8.2–13%, and the majority of the patients in these studies exhibited ST-elevation myocardial infarction (STEMI) treated with primary percutaneous coronary intervention (PCI). In contrast, there are limited data available regarding the prevalence of UMI in patients with non-ST-elevation acute coronary syndrome (NSTE-ACS). Currently, although coronary computed tomography angiography (CCTA) has not been recommended as a risk-stratifying tool of ACS, recent reports supported the clinical potentials and implications of CCTA in the ACS setting (8, 9). Therefore, in this study, we sought to assess the prevalence of non-infarct-related territory UMI (non-IR UMI) in patients presenting with a first episode of NSTE-ACS without a history of MI, PCI, or coronary artery bypass graft (CABG). We further evaluated the association between the presence of non-IR UMI and CCTA findings including qualitative and quantitative assessments including the stenosis severity, Agatston score, peri-coronary fat attenuation index (FAI), epicardial fat volume (EFV) and epicardial fat attenuation (EFA) to assess if CCTA findings including peri-coronary adipose tissue and pericardial fat assessments could predict the presence of non-IR UMI before invasive coronary angiography.

## Materials and Methods

### Study Design and Patient Population

**Figure 1:**
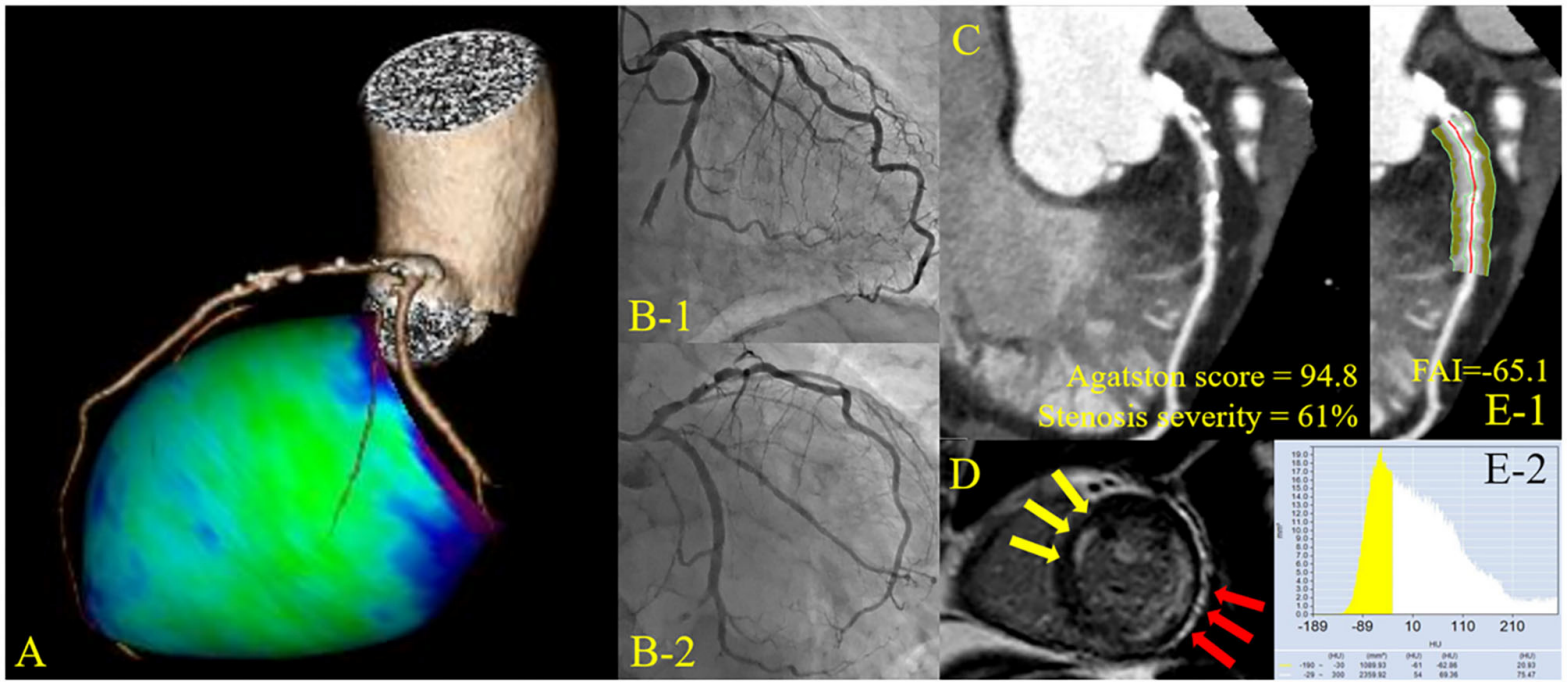
Representative cardiac images of a patient with non-infarct-related unrecognized myocardial infarction. A 72-year-old man with a history of diabetes mellitus. **(A)** Three-dimensional reconstruction of the coronary artery. **(B)** Pre-percutaneous coronary intervention (PCI) angiogram reveals 99% stenosis in the left circumflex artery #13 (culprit lesion) and 75% stenosis in the left anterior descending artery #6 (non-culprit lesion). **(C)** Curved planar reconstruction view with Agatston score and computed tomography-derived stenosis severity. **(D)** Delayed enhancement cardiac magnetic resonance imaging shows infarct-related late gadolinium enhancement (LGE) in the lateral territory and non-infarct-related LGE in the anterior territory. **(E)** Peri-coronary fat attenuation index (FAI) at the proximal 40 mm segment of non-infarct-related vessel (left anterior descending artery) was traced.

This study was a retrospective analysis of prospectively, but non-consecutively enrolled patients in the institutional NSTE-ACS CCTA research registry at Tsuchiura Kyodo General Hospital, which tested the hypothesis that CCTA before invasive coronary angiography may provide the diagnostic and therapeutic information of atherosclerotic burden, lesion location and procedural planning of revascularization in patients with suspected NSTE-ACS. From this registry data, 169 patients with the first episode of NSTE-ACS who underwent subsequent coronary angiography with *ad-hoc* uncomplicated PCI and cardiac magnetic resonance imaging (CMR) within 30 days after the index PCI and before non-IR significant lesion staged PCI were enrolled in the present study. Uncomplicated PCI was defined as post-PCI Thrombolysis in Myocardial Infarction flow grade ≥3, residual angiographic diameter stenosis less than 20%, no side branch occlusion with a diameter more than 1.5 mm or visible distal embolization, and no PCI-related myocardial infarction according to current guidelines (10). Diagnosis of NSTE-ACS was made by symptoms, biomarker elevation, and ECG changes. Patients with NSTE-ACS were enrolled according to the ESC guidelines for the management of acute coronary syndromes in patients presenting without persistent ST-segment elevation (11). We included patients aged at least 20 years who were admitted to the intensive care unit with a diagnosis of NSTE-ACS within 48 h of the last appearance of symptoms suggestive of myocardial ischemia and/or ST-T segment change in at least 2 leads. All patients subsequently underwent uncomplicated PCI with an early invasive strategy less than 48 h after admission (12). We included patients with unstable angina pectoris (UAP) and non–ST-elevation myocardial infarction (NSTEMI) when the single culprit lesion was identifiable and considered suitable for PCI. We excluded patients with significant left main coronary artery disease (CAD), chronic total occlusion, unidentifiable culprit lesions, significant valvular disease, previous CABG, previous MI, significant arrhythmia, renal insufficiency with a baseline serum creatinine level >1.5 mg/dL, and contraindication to CMR (eg, pacemaker, internal defibrillator, or other incompatible intracorporeal foreign bodies, pregnancy, and claustrophobia). In the case of multivessel disease, delayed enhancement (DE)-CMR imaging was performed before staged non-IR lesion revascularization. Patients treated with multivessel PCI for not only culprit but non-culprit territory lesions at the index urgent PCI were excluded from the final analysis. A representative case undergoing urgent PCI with preprocedural CCTA and postprocedural CMR is shown (Figure 1). The study protocol agreed with the Declaration of Helsinki and was approved by the institutional ethics committee (TKGH-IRB 2021FY85). All patients provided written informed consent for future enrollment in the institutional clinical studies. Prompt optimal medical therapy was initiated in all patients before urgent PCI and guideline-directed medical therapy was continued thereafter.

### Urgent Coronary Angiography and PCI

Invasive coronary angiography (CAG) and revascularization of the IR lesion were performed by *ad-hoc* PCI via the routine use of drug-eluting stents with a 6-French system. Before the PCI procedure, all patients received a loading dose of 200 mg aspirin and 300 mg clopidogrel or 20 mg prasugrel. Coronary angiograms were analyzed quantitatively using QAngio XA system (Medis Medical Imaging Systems, The Netherlands). The IR lesion was identified by the combination of electrocardiography (ECG), echocardiography, and coronary angiographic findings by two expert interventionalists. The stent type and procedure strategy selected were at the operator's discretion.

### CCTA Acquisition

CCTA imaging was performed before PCI, indicating that the median value of the interval between CCTA and PCI was 4.3 (2.1–7.2) h. Computed tomography (CT) imaging was performed using a 320-slice CT scanner (Aquilion ONE; Canon Medical Systems Corporation, Japan) in accordance with the Society of Cardiovascular Computed Tomography guidelines (13) in all patients. All CCTA examinations were performed using a single CT system during the present study period. When needed, oral and/or intravenous beta-blockers were administered to achieve a target heart rate ≤ 65 bpm. A non-contrast enhanced CT-scan for the assessment of coronary artery calcification, prospectively triggered at 75% of the RR-interval with 3 mm slice thickness, was followed by CCTA. Immediately before CCTA scanning, 0.3 or 0.6 mg of sublingual nitroglycerine was administered. The scan was triggered using an automatic bolus-tracking technique with a region of interest placed in the ascending aorta. Images were acquired after a bolus injection of 40 to 60 mL contrast (iopamidol, Bayer Yakuhin, Ltd., Japan) at a rate of 3–6 mL/s, using prospective ECG-triggering or retrospective ECG gating with tube current modulation. Acquisition and reconstruction parameters for the patients were 120 kVp, tube current of 50 to 750 mA, gantry rotation speed of 350 ms per rotation, helical pitch of 8 to 18, field matrix of 512 × 512, and scan thickness of 0.5 mm. All scans were performed during a single breath-hold. Images were reconstructed at a window centered at 75% of the R-R interval to coincide with left ventricular diastasis.

### Analyses of FAI, Epicardial Fat, CCTA Plaque, and Agatston Score

In the present study, the crude analysis of FAI of all three main coronary vessels was performed. The mean FAI of three main coronary vessels or the vessel specific FAI value was used for the analysis. FAI analysis was performed in the proximal 40 mm segments of left anterior descending coronary artery and left circumflex coronary artery and the proximal 10 to 50 mm segment of the right coronary artery using a dedicated workstation Aquarius iNtuition Edition version 4.4.13; TeraRecon Inc., USA), as previously described (14, 15). Within the pre-identified segment of interest, the lumen as well as the inner and outer vessel wall border were tracked in an automated manner with additional manual optimization. Adipose tissue was defined as all voxels with attenuation between −190 HU and −30 HU. The FAI value was defined as the average CT attenuation in HU of the adipose tissue located within a radial distance from the outer vessel wall equal to the diameter of the coronary vessel (Figure 1).

EFV and EFA was quantified offline on non-contrast CT images in all patients, using a semiautomatic software equipped in the dedicated workstation (AZE Virtual Place, Canon Medical Systems Corporation, Japan). Region of interest (ROI) were drawn by manual tracing of the pericardium in axial planes from the take-off of right pulmonary artery to apex of the heart. Then, EFV and EFA were automatically calculated as the sum of all pixels within a window of −190 and −30 HU in the ROI. FAI and epicardial fat analysis were separately performed as a *post-hoc* analysis blinded to CMR results at the institutional imaging and physiology laboratory by the expert investigators for FAI analysis (KM and M. Hoshino). Plaque assessment on CCTA was independently performed by two experienced readers (M. Hada and TM) using the reconstructed CCTA images transferred to an offline workstation (Ziostation2, Ziosoft Inc., Japan).

Coronary artery calcium (CAC) quantification was performed on non-contrast CT images by Agatston method using the software facilitating semi-automatic assessment (Ziostation2, Ziosoft Inc., Japan). This software can allow the user to relate calcification to the specific coronary arteries by locating area with attenuation ≥ 130HU.

### CMR Examination

#### CMR Acquisition and Assessment of IR (ACS Culprit) Scar and Non-IR UMI

Images were acquired on a 1.5-T scanner (Philips Achieva, Philips Medical Systems, The Netherlands) with 32-channel cardiac coils within 30 (8–32) days after the IR lesion PCI and before the staged PCI of non-IR significant lesions. Cardiac gating and heart rate recording were achieved using the vector-cardiogram device. Blood pressure and heart rate were monitored throughout the protocol. Cine-CMR was performed using a retrospectively gated steady-state free precession sequence. Twelve short-axis slices of the left ventricle (LV) were acquired from the apex to the base. The cine-CMR parameters were as follows: repetition time/echo time, 4.1 ms/1.4 ms; slice thickness, 6 mm; flip angle, 55°; field of view, 350 × 350 mm^2^; matrix size, 128 × 128; and number of phases per cardiac cycle, 20. LV mass and volumes were calculated according to the Simpson's rule using CMR data (16). Gadolinium contrast was infused intravenously at a total dose of 0.10 mmol/kg. Fifteen min after this injection, LGE images were acquired in the same planes as cine images and imaging parameters were as follows: repetition time/echo time, 3.8 ms/1.28 ms; flip angle, 15°; field of view, 350 × 350 mm^2^; acquisition matrix, 200 × 175; number of phases per cardiac cycle, and slice thickness, 8 mm. The infarcted myocardium was quantified on the LGE images as myocardium with a signal intensity exceeding the mean signal intensity of the remote myocardium by >5 standard deviation (SD) and using a semi-automatic algorithm. Non-IR UMI was defined as absence of MI/PCI/CABG history on medical records, but the presence of LGE on the non-IR territories by a consensus of two experienced cardiologists (KS and YK) and controlled by an expert reader (TK) masked to the patient data. High signal intensity lesion located in subendocardial territories with a ischemic distribution consistent of specific epicardial coronary arteries on LGE images were considered to represent non-IR UMI or IR scar. Infarct size of both ACS (IR) and UMI (non-IR) was measured using the full width at one-half maximum method. Microvascular obstruction (MVO) was defined as the hypo-enhanced region within and included in the infarcted myocardium. LV mass was normalized to body surface area as LV mass index (LVMI). All CMR images were analyzed using dedicated off-line software (AZE Virtual Place, Canon Medical Systems Corporation, Japan).

### Statistical Analysis

Statistical analyses were performed using SPSS version 25.0 (IBM Corporation, USA) and R version 4.0.3 (The R Foundation for Statistical Computing, Austria) software. Both patient-based and vessel-based analyses were tried to identify the predictors of non-IR UMI. Categorical data were expressed as numbers and percentages and compared by the chi-square or Fisher's exact tests. Continuous data were expressed as median (interquartile range [IQR]) and analyzed using Mann–Whitney U test. Receiver operating curves were analyzed to assess the best cut-off values of significant predictors for the presence of non-IR UMI using CCTA variables. The optimal cut-off value was calculated using the Youden index.

Univariable and multivariable linear regression analyses were performed to determine predictive factors of non-IR UMI size. Univariable and multivariable logistic regression analyses were performed to predict the presence of non-IR UMI using CCTA variables. The covariates with *p* < 0.10 in the univariable analysis were included in the multivariable analysis. A collinearity index was used for checking linear combinations among covariates, and Akaike information criterion for avoiding overfitting. The Generalized estimating equations approach was used to take into account the within-subject correlation due to multiple vessels analyzed within a single patient.

The prediction model for non-IR UMI was constructed to determine the incremental discriminatory and reclassification performance of FAI, by using relative integrated discrimination improvement (IDI) and category-free net reclassification index (NRI) when FAI was added to the clinical risk model including GRACE score, ejection fraction (EF), Agatston score, and CCTA-derived stenosis severity. A two-sided *p* < 0.05 was considered statistically significant.

## Results

### Baseline Patient Characteristics, CCTA and CMR Findings

Of 169 initially enrolled patients, 5 and 6 patients were excluded from the final analysis because of unsatisfactory CCTA and DE-CMR data acquisition, respectively. Thus, the final analysis was performed on 158 patients with complete pre-PCI CCTA and post-PCI DE-CMR data. Of 158 patients for the final analysis, 8 non-IR lesions were treated at the index urgent PCI of NSTE-ACS. These vessels were excluded from the vessel-based UMI assessment because PCI-related myocardial injury could not be differentiated from that by UMI-related scar. Therefore, the vessel-based analysis was performed in 308 vessels and territories on CCTA after excluding 8 territories from the vessel-based non-IR UMI analysis. The patients' clinical characteristics, CCTA and CMR findings according to the presence or absence of non-IR UMI are presented in Tables 1, 2. In 158 patients, median age was 66 (58–72) years, and 76.6 % were men. Non-IR UMI was detected in 30 territories of 28 (17.7%) patients, two of which had multiple non-IR-UMI on two different territories. The patients with non-IR UMI, when compared with those without non-IR UMI, showed significantly lower EF. The prevalence of non-IR UMI was significantly higher in patients with NSTEMI in comparison with those with UAP [27/129 (20.9%) vs. 1/29 (3.4%), *P* = 0.029]. On the vessel-based analysis of CCTA, non-IR UMI was significantly associated stenosis severity represented by CCTA-derived area stenosis and quantitative coronary angiography (QCA)-derived diameter stenosis. Vessels with non-IR UMI showed significantly higher total Agatston scores and the vessel-specific Agatston score. Of note, the vessel-specific FAI values of vessels with non-IR UMI tended to be higher than those without. On a patient-based analysis, the mean FAI values were significantly elevated in patients with non-IR UMI (Table 2). EFV and EFA showed no significant association with the presence of non-IR UMI, for both patient-based and vessel-based analyses. The degree of IR myocardial injury post PCI evaluated by creatine kinase (CK)-MB was significantly higher in patients with non-IR UMI than in those without unrecognized non-IR UMI (*p* = 0.043). Furthermore, the degree of IR-LGE was weakly albeit significantly correlated with the degree of non-IR LGE (r = 0.204, *p* = 0.010).

**Table 1:** Baseline characteristics.

	**Total**	**Patients with non-IR UMI**	**Patients without non-IR UMI**	***P* value**
	**(*n =* 158)**	**(*n =* 28)**	**(*n =* 130)**	
Age (years)	66 (58 to 72)	65 (59 to 72)	66 (58 to 72)	0.819
Male, *n* (%)	121 (76.6%)	28 (80.0%)	97 (75.2%)	0.658
Hypertension, *n* (%)	110 (69.6%)	20 (66.7%)	90 (70.3%)	0.667
Diabetes mellitus, *n* (%)	50 (31.7%)	11 (36.7%)	39 (30.5%)	0.519
Hyperlipidemia, *n* (%)	96 (60.8%)	14 (46.7%)	82 (64.1%)	0.097
Smoker, *n* (%)	70 (44.3%)	16 (53.3%)	54 (41.1%)	0.310
LDL cholesterol (mg/dl)	120 (104 to 151)	112 (95 to 140)	122 (105 to 152)	0.673
eGFR (ml/min/1.73m^2^)	75.1 (62.2 to 84.8)	66.6 (55.5 to 79.6)	75.3 (64.1 to 85.4)	0.117
EF (%)	62.0 (55 to 66)	55 (51 to 60)	63 (58 to 66)	<0.001
Peak Troponin (ng/l)	3,269 (730 to 14869)	6,269 (2162 to 22204)	2,565 (674 to 12362)	0.172
Peak CK (U/l)	250 (129 to 655)	345 (174 to 983)	247 (120 to 608)	0.339
Peak CK-MB (U/l)	24 (12 to 56)	38 (18 to 58)	22 (12 to 55)	0.043
CRP (mg/dl)	0.14 (0.06 to 0.60)	0.25 (0.11 to 0.95)	0.13 (0.05 to 0.54)	0.191
GRACE score	115 (95 to 146)	124 (104 to 149)	113 (93 to 144)	0.078
SYNTAX score	12 (8 to 20)	16 (11 to 23)	11 (8 to 19)	0.054
Lesion location				
RCA culprit, *n* (%)	42 (26.6%)	4 (14.3%)	38 (29.2%)	0.156
LAD culprit, *n* (%)	79 (50.0%)	16 (57.1%)	63 (48.5%)	0.533
LCX culprit, *n* (%)	37 (23.4%)	8 (28.6%)	29 (22.3%)	0.469
Multivessel disease, *n* (%)	49 (31.0%)	11 (39.3%)	38 (29.2%)	0.368
Clinical status				0.029
NSTEMI, *n* (%)	129 (81.6%)	27 (20.9%)	102 (78.5%)	
UAP, *n* (%)	29 (18.4%)	1 (3.4%)	28 (96.6%)	

*Values are n (%) or median (interquartile range). Non-IR UMI, non-infarct-related unrecognized myocardial infarction; LDL, low density lipoprotein; eGFR, estimated glomerular filtration rate; EF, ejection fraction; CK, creatine kinase; CK-MB, creatine kinase-MB; CRP, C-reactive protein; RCA, right coronary artery; LAD, left anterior descending coronary artery; LCX, left circumflex coronary artery; NSTEMI, non-ST-elevation myocardial infarction; UAP, unstable angina pectoris*.

**Table 2:** QCA, CCTA, and CMR findings.

	**Total**	**Vessels with non–IR UMI**	**Vessels without non–IR UMI**	***P* value**
	**(*n =* 308)**	**(*n =* 30)**	**(*n =* 278)**	
**QCA**				
Diameter stenosis (%)	27.3 (14.7 to 46.6)	51.3 (27.1 to 73.1)	26.5 (14.6 to 41.9)	<0.001
MLD (mm)	2.16 (1.47 to 2.78)	1.85 (0.96 to 2.79)	2.18 (1.51 to 2.78)	0.185
**CCTA**				
Plaque features				
Low attenuation plaque, *n* (%)	40 (13.0%)	4 (13.3%)	36 (12.9%)	>0.999
Napkin ring sign, *n* (%)	5 (1.6%)	1 (3.3%)	4 (1.4%)	0.424
Spotty calcification, *n* (%)	118 (38.3%)	11 (36.7%)	107 (38.5%)	0.704
Positive remodeling ≥ 1.10, *n* (%)	62 (20.1%)	6 (20.0%)	56 (20.1%)	>0.999
Remodeling index	0.94 (0.83 to 1.06)	0.98 (0.82 to 1.05)	0.94 (0.83 to 1.06)	0.446
Agatston score				
Agatston score total	217 (61 to 714)	356 (180 to 1047)	202 (56 to 659)	0.015
Agatston score in each vessel	135 (34 to 135)	57 (32 to 171)	30 (0 to 134)	0.034
Agatston score of non-IR vessels average	49 (14 to 212)	91 (42 to 309)	43 (6 to 157)	0.043
Epicardial fat				
Epicardial fat attenuation (HU)	–77.3 (–80.6 to –73.5)	–75.6 (–79.2 to –73.9)	–77.7 (–80.7 to –73.5)	0.340
Epicardial fat volume (cm^3^)	126 (96 to 167)	118 (95 to 161)	125 (96 to 167)	0.700
FAI				
FAI average (HU)	–69.5 (–74.0 to –64.5)	–64.9 (–71.5 to –60.3)	–69.7 (–74.4 to –64.7)	0.005
FAI in each vessel (HU)	–69.4 (–75.0 to –63.1)	–68.2 (–72.8 to –59.8)	–69.6 (–75.3 to –63.5)	0.078
CT*-*derived stenosis				
CT-derived stenosis severity (%)	35.9 (28.0 to 45.0)	43.6 (32.7 to 100.0)	35.0 (28.5 to 44.0)	<0.001
MLA (mm^2^)	4.2 (2.9 to 6.4)	2.9 (0.0 to 4.9)	4.3 (2.9 to 6.6)	0.001
**CMR**				
Total LGE volume (ml)	2.7 (0.0 to 7.3)	7.8 (2.9 to 14.1)	2.4 (0.0 to 6.2)	<0.001
IR LGE volume (ml)	1.9 (0.0 to 5.6)	2.7 (0.7 to 5.2)	1.9 (0.0 to 5.6)	0.694
Non-IR LGE volume (ml)	0.0 (0.0 to 0.0)	3.2 (1.4 to 5.0)		
Non-IR RCA LGE volume (ml)	0.0 (0.0 to 0.0)	2.8 (0.9 to 4.6)		
Non-IR LAD LGE volume (ml)	0.0 (0.0 to 0.0)	2.3 (0.7 to 3.6)		
Non-IR LCX LGE volume (ml)	0.0 (0.0 to 0.0)	5.1 (4.4 to 8.4)		

*Values are n (%) or median (interquartile range). QCA, quantitative coronary angiography; CCTA, coronary computed tomography angiographic; CMR, cardiac magnetic resonance; UMI, unrecognized myocardial infarction; MLD, minimal lumen diameter; non-IR, non-infarct-related; FAI, peri-coronary fat attenuation index; CT, computed tomography; MLA, minimum lumen area; LGE, late gadolinium enhancement; IR, infarct-related; RCA, right coronary artery; LAD, left anterior descending coronary artery; LCX, left circumflex coronary artery*.

### Determinant of the Presence of Non-IR UMI on CCTA

**Figure 2:**
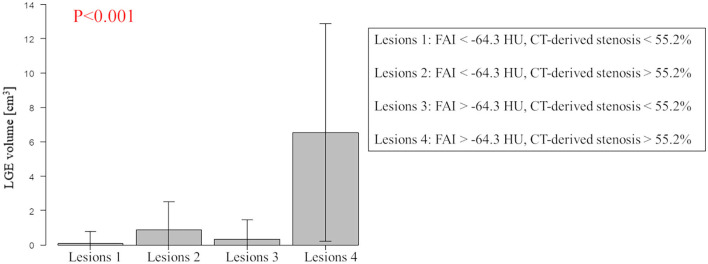
Non-IR UMI volume stratified by the best cut-off values of FAI and CT-derived stenosis severity. Lesions with FAI >-64.3 and CT-derived stenosis >55.2% showed higher non-IR LGE volume (non-IR UMI volume). Non-IR, non-infarct-related; UMI, unrecognized myocardial infarction; CT, computed tomography; LGE, late gadolinium enhancement; FAI, peri-coronary fat attenuation index.

**Figure 3:**
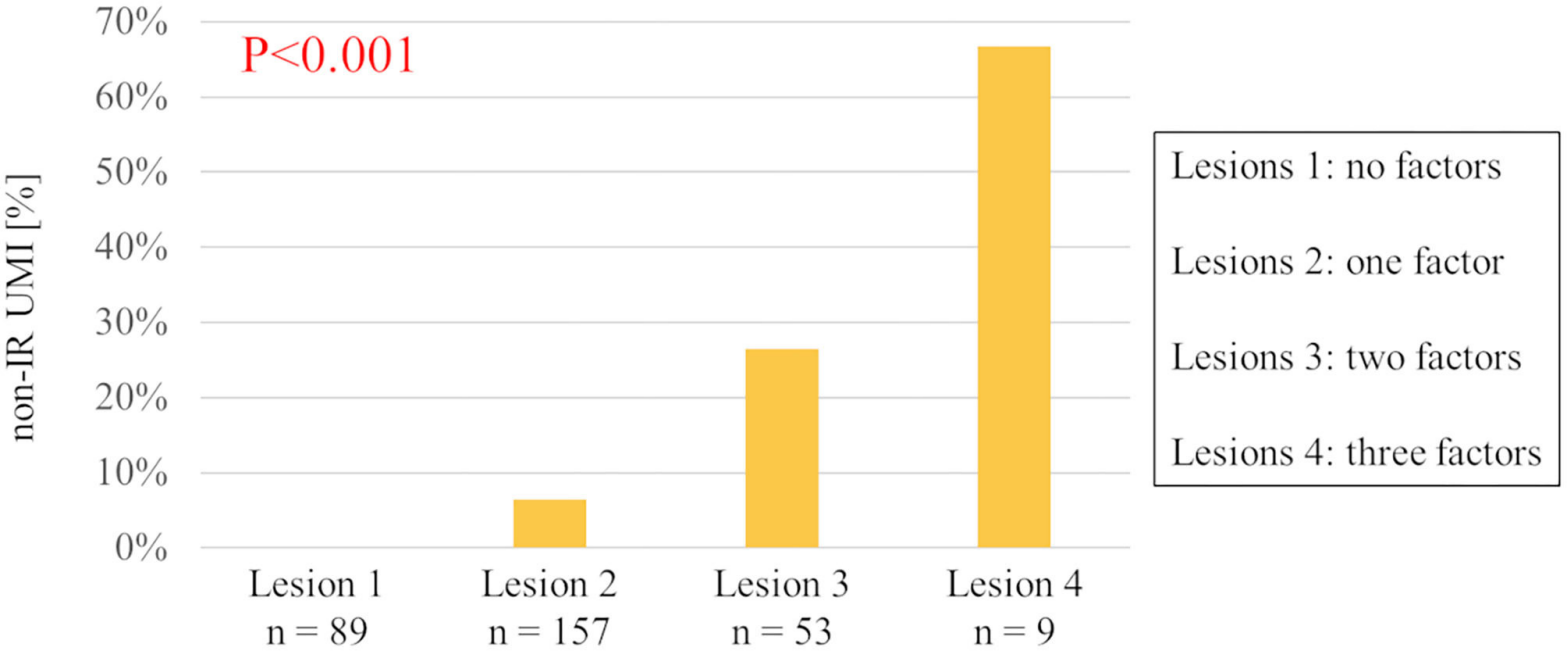
The prevalence of non-IR UMI stratified by the numbers of three predictive CCTA features. Relevant CCTA factors assessed are (1) peri-coronary fat attenuation index >-64.3, (2) Agatston score >30.0, and (3) CCTA-derived stenosis >55.2%. Non-IR UMI, non-infarct-related unrecognized myocardial infarction; CCTA, coronary computed tomography angiographic.

The results of univariable and multivariable logistic regression analyses to determine the predictors of the presence of non-IR UMI are presented in Table 3. When using CCTA variables, stenosis severity, Agatston score, and mean FAI were independently predictive of the presence of non-IR UMI. The best cut-off values of the FAI and Agatston score to predict the presence of non-IR UMI were −64.3 and 30.0, respectively. Multivariable linear regression analysis revealed that FAI and CT-derived stenosis were independent predictors of non-IR LGE volume (Table 4). For the prediction of non-IR UMI volume, Figure 2 shows the non-IR UMI volume stratified by the best cut-off values of FAI (-64.3) and CT-derived stenosis severity (55.2%). For the presence or absence of non-IR UMI, the prevalence stratified by the numbers of significant CCTA predictors ([1] FAI >-64.3, [2] Agatston score >30.0, and [3] CT-derived stenosis >55.2%) was presented in Figure 3. The discriminant efficacy (IDI and NRI) of predicting non-IR UMI was significantly improved when FAI was added to the clinical risk model (Table 5).

**Table 3:** Univariable and multivariable logistic analysis of predicting non-IR UMI.

	**Univariable analysis**			**Multivariable analysis**		
	**OR**	**95% CI**	***P* value**	**OR**	**95% CI**	***P* value**
Age	1.00	0.97 to 1.04	0.963			
Male	1.12	0.43 to 2.90	0.812			
EF	0.94	0.92 to 0.97	<0.001			not selected
Agatston score of non-IR vessels average	1.00	1.00 to 1.00	0.018			
Agatston score of non-IR vessels average >30.0	12.06	2.86 to 50.84	0.001	8.39	2.17 to 32.45	0.002
FAI average	1.09	1.02 to 1.17	0.012			
FAI average >-64.3	3.32	1.55 to 7.12	0.002	3.23	1.34 to 7.81	0.009
MLA	0.76	0.60 to 0.96	0.021			
CT-derived stenosis	1.05	1.03 to 1.07	<0.001	1.04	1.02 to 1.06	<0.001
QCA-derived diameter stenosis	1.04	1.03 to 1.06	<0.001			not selected
SYNTAX score	1.04	1.01 to 1.07	0.011			
GRACE score	1.01	1.00 to 1.03	0.043			not selected
NSTEMI	7.32	0.99 to 53.98	0.051			

*Non-IR, non-infarct-related; UMI, unrecognized myocardial infarction; OR, odds ratio; CI, confidence interval; EF, ejection fraction; FAI, peri-coronary fat attenuation index; MLA, minimum lumen area; CT, computed tomography; QCA, quantitative coronary angiography; NSTEMI, non-ST-elevation myocardial infarction*.

**Table 4:** Univariable and multivariable linear regression analysis of predicting non-IR LGE volume.

	**Univariable analysis**			**Multivariable analysis**		
	**β**	**95% CI**	***P* value**	**β**	**95% CI**	***P* value**
Age	−0.010	–0.028 to 0.009	0.326			
Male	0.014	–0.491 to 0.462	0.952			
EF	–0.043	–0.064 to –0.022	<0.001	–0.011	–0.032 to 0.011	0.341
Agatston score of non-IR vessels average	0.001	0.000 to 0.002	0.015			
Agatston score of non-IR vessels average >30.0	0.443	0.033 to 0.852	0.034	–0.057	–0.459 to 0.345	0.779
FAI average	0.046	0.015 to 0.077	0.003			
FAI average >–64.3	1.007	0.544 to 1.469	<0.001	0.666	0.209 to 1.124	0.004
MLA	–0.137	–0.204 to –0.069	<0.001			
CT*-*derived stenosis	0.042	0.032 to 0.052	<0.001	0.037	0.026 to 0.049	<0.001
QCA-derived diameter stenosis	0.031	0.022 to 0.040	<0.001			
SYNTAX score	0.019	–0.003 to 0.041	0.087			
GRACE score	0.011	0.005 to 0.016	<0.001	0.004	–0.002 to 0.009	0.124
NSTEMI	0.458	–0.062 to –0.977	0.084			

*Non-IR, non-infarct-related; LGE, late gadolinium enhancement; CI, confidence interval; EF, ejection fraction; FAI, peri-coronary fat attenuation index; MLA, minimum lumen area; CT, computed tomography; QCA, quantitative coronary angiography; NSTEMI, non-ST-elevation myocardial infarction*.

**Table 5:** Prediction models for the presence of non-IR UMI.

**Prediction model**	**C-statistics**	***P* value**	**IDI**	***P* value**	**NRI**	***P* value**
Clinical model 1	0.783	–	Reference	–	Reference	–
Clinical model 2	0.846	0.008	0.062	0.015	0.583	0.002

*Clinical model 1 (EF + GRACE score + Agatston score + CT-derived stenosis). Clinical model 2 (EF + GRACE score + Agatston score + CT-derived stenosis + FAI). Non-IR UMI, non-infarct-related unrecognized myocardial infarction; IDI, integrated discrimination improvement; NRI, net reclassification index; EF, ejection fraction; CT, computed tomography; FAI, peri-coronary fat attenuation index*.

## Discussion

This study investigated the prevalence of non-IR UMI and its CCTA-derived predictors in patients with a first clinical episode of NSTE-ACS. The main findings of this study are summarized as follows: (1) the prevalence of non-IR UMI was 17.7 %; (2) on CCTA performed before urgent PCI, non-IR UMI was significantly associated with EF on the patient-based analysis; (3) the median volume of non-IR UMI was 3.2 (1.4–5.0) ml; (4) on the vessel-based analysis, non-IR UMI was significantly associated with CT-derived stenosis severity, Agatston score, and FAI; (5) peak cardiac marker elevation (CK-MB) post PCI was significantly higher in patients with non-IR UMI compared with those without non-IR UMI; (6) on multivariable linear regression model, vessel-based FAI and CT-derived stenosis were significant predictors of non-IR UMI volume; (7) FAI significantly improved NRI and IDI over the reference prediction model including GRACE score, EF, CCTA-derived stenosis severity, and Agatston score.

Previous studies reporting the prevalence of CMR-derived non-IR UMI in patients with ACS are scarce and this is the first study to investigate CCTA-derived predictors of non-IR UMI by DE-CMR and its prevalence in patients with a first clinical episode of NSTE-ACS. The presence of unrecognized myocardial scar detected by LGE in patients presenting with first acute AMI has been reported to be 8.2–13.0% (6, 7). The majority of the patients in these studies exhibited STEMI treated with primary PCI. In contrast, our results indicated that the prevalence of UMI in NSTE-ACS was higher than these previous reports.

Currently, CCTA is a class I (level of evidence A) recommendation as an alternative to coronary angiography for exclusion of ACS in patients at low-to-intermediate risk of CAD with suspected acute coronary syndrome to confirm the diagnosis and evaluate the risk of future events (11). Although CAC quantification on non-contrast CT is widely acknowledged as an important decision tool for identifying stable patients with coronary atherosclerosis who would benefit from preventive treatments, it is not used as a gatekeeper in patients with chest pain in the emergency department. Our results suggested that Agatston score-defined CAC on CCTA in the setting of NSTE-ACS may help risk stratify high-risk patients with non-IR UMI in combination of FAI assessment independent of stenosis assessment and high-risk plaque features. Further studies are needed to test if the prediction of non-IR UMI could be associated with worse prognosis after PCI in patients with a first clinical episode of NSTE-ACS.

### Association of Agatston Score, Stenosis Severity, and FAI With Non-IR UMI

Agatston score-defined CAC quantification has emerged as one of the most reliable ways to estimate cardiovascular risk (17). Although the exact pathophysiological mechanisms causing UMI remain to be determined, our results agreed with the previous studies which demonstrated a significant association of angiographic stenosis severity and Agatston score with the presence of UMI (18, 19) in patients with stable CAD. Agatston score and stenosis severity could be the signature of the advanced atherosclerotic burden, and the presence of UMI might represent the inter-relationship of the high-risk patient characteristics. The progression of atherosclerosis including plaque healing after asymptomatic rupture or erosion may lead to distal thrombosis and an increase in plaque burden and stenosis progression, resulting in the potential occurrence of UMI in the subtended territory (20–22).

It has been recently reported that the mean peri-coronary adipose tissue attenuation value expressed by FAI on CCTA is associated with cardiac mortality (14). FAI, a marker of perivascular inflammation, was significantly associated with the presence of non-IR UMI in this study. This finding may suggest that local inflammation caused by plaque instability could link with the occurrence of non-IR UMI. Recent studies reported the relationship between FAI and CCTA-derived unstable plaque features (23, 24). These vulnerable plaques with high inflammation may occasionally cause rupture or erosion, leading to debris liberation, distal embolization of the myocardium, and the occurrence of UMI. Recent studies have focused on the detection of vascular inflammation by using non-invasive imaging modalities such as positron emission tomography imaging (25, 26). A recent study reported that FAI was associated with 18F-fluorodeoxyglucose uptake (27), indicating the feasibility of CT attenuation of peri-coronary adipose tissue for detecting peri-coronary inflammation. Our study supports this notion and extends that vascular inflammation could be linked with non-IR UMI in patients with a first clinical episode of NSTE-ACS. Given the well-accepted association between atherosclerosis and inflammation, and the recently reported CANTOS study (28) and COLCOT trial (29) showing the link with anti-inflammatory therapy and the reduction of recurrent cardiovascular events, further studies are needed to test our hypothesis generating results and if anti-inflammatory intervention could reduce the FAI values and the occurrence of UMI.

### Clinical Implications

Recent reports supported the clinical potentials and implications of CCTA in the ACS setting (8, 9). Previous investigations have suggested that the presence of UMI confers an increased risk of adverse events (2, 3, 7). Recently, the presence of unrecognized myocardial scar detected by LGE in patients presenting with first acute AMI has been reported to be associated with worse outcomes (6, 7). Although the current study lacks prognostic information, our findings suggest that CCTA in the setting of NSTE-ACS could provide the information of the previous occurrence of non-IR UMI and the status of vascular inflammation, potentially resulting in the risk stratification in these patients showing a wide heterogeneity in its symptoms and prognosis reported in patients with NSTE-ACS (30, 31). A recent study by Antiochos et al. reported, in a multicenter cohort study of patients with suspected coronary artery disease, that presence of UMI or clinically recognized MI portended an equally significant risk for death and MI, independently of the presence of ischemia (2). Further studies are required to test the clinical significance of CCTA in the setting of NSTE-ACS, particularly with respect to the inflammatory status by FAI and the predictability of non-IR UMI and its prognostication.

### Limitations

This study was a single center analysis of prospectively enrolled from the registry data and pertains an observational nature, and its inherent limitation exists. Rigorous exclusion criteria and the CMR protocol limited the number of study patients and may have resulted in a certain level of selection bias. Patients were screened after knowing the contraindications to CMR and the importance of ECG gating, leading to further selection bias, because we had no patients with metallic device implants, bronchospasm, claustrophobia, or atrioventricular block. Because patients are more likely to present with multivessel coronary artery disease in NSTE-ACS, compared with those with STEMI, determining the IR vessel in patients with NSTE-ACS can be challenging. The final call was discretion of the operator and consensus reading of two expert interventionalists, which was an important limitation of the present study. Although this study included a relatively large number of patients with pre-PCI CCTA and post-PCI CMR data, the present sample size still limited the confidence of the overall statistical analyses, as well as extensive subgroup analyses. The assessment of involved myocardial segments by a coronary stenosis was determined by the coronary anatomy, and no objective method was applied, although there are no universally accepted criteria for this purpose. Finally, this study lacks prognostic information and further large sample studies are needed to test the results obtained from the present study.

## Conclusion

In patients with a first clinical episode of NSTE-ACS who underwent urgent PCI, the prevalence of non-IR UMI was 17.7 %. The comprehensive CCTA assessment including FAI, Agatston score, and stenosis severity could identify patients with non-IR UMI before invasive CAG. Our findings warrant further investigation to test if comprehensive CCTA assessment for the presence of non-IR UMI may provide prognostic information or guide intensive therapeutic strategy.

## Data Availability Statement

The raw data supporting the conclusions of this article will be made available by the authors, without undue reservation.

## Ethics Statement

The studies involving human participants were reviewed and approved by Tsuchiura Kyodo General Hospital Ethics Committee. The patients/participants provided their written informed consent to participate in this study.

## Author Contributions

KM, MHo, and TK contributed to conception and design of the study. KM and MHo organized the database, performed the statistical analysis, and wrote the first draft of the manuscript. All authors contributed to manuscript revision, read, and approved the submitted version.

## Conflict of Interest

The authors declare that the research was conducted in the absence of any commercial or financial relationships that could be construed as a potential conflict of interest.

## Publisher's Note

All claims expressed in this article are solely those of the authors and do not necessarily represent those of their affiliated organizations, or those of the publisher, the editors and the reviewers. Any product that may be evaluated in this article, or claim that may be made by its manufacturer, is not guaranteed or endorsed by the publisher.
